# Recommending the Addiction Severity Index for Swedish Social Services: Where is the Evidence?

**DOI:** 10.1177/14550725261498138

**Published:** 2026-10-25

**Authors:** Alexander Björk

**Affiliations:** 1 Department of Social Work, 7675 Stockholm University, Stockholm, Sweden

**Keywords:** addiction severity index, Sweden, social services, clinical practice guidelines, evidence

## Abstract

The article argues that there is a lack of clear evidence that motivates the strong recommendation of the standardized assessment instrument Addiction Severity Index (ASI) in the Swedish National Board of Health and Welfare's (NBHW) national practice guidelines. The argument centers on the municipal social services sector where the ASI has gained most traction. The article reviews the assessments underpinning the NBHW's recommendation of the ASI, points out unclear links between presented evidence and recommendations, and calls attention to research that is omitted from the guidelines. Finally, implications and possible ways forward regarding the social services' assessment of clients with substance use problems are discussed.

## Introduction

The Addiction Severity Index (ASI) is a standardized assessment instrument widely used in research as well as in clinical settings, both in Sweden and internationally (McLellan et al., 2006). It was launched in the USA in the 1970's as a “semistructured clinical research interview designed to measure patient status in seven functional domains: alcohol and drug use, medical and psychiatric health, employment/self support, family relations, and illegal activity” (McLellan et al., 2006, p. 113). It comprises an intake and a follow-up interview, encompassing 180 and 140 questions, respectively, in the Swedish version. In addition to providing outcome measures for treatment research, the ASI has been presented as a clinical tool that can help professionals assess and follow-up clients.

Clinical practice guidelines in the Nordic countries have issued evidence-based recommendations concerning the use of the ASI in professional practice, and the Swedish guidelines stand out in strongly recommending the ASI. Whereas the Swedish guidelines recommend the ASI at the highest priority (National Board of Health and Welfare [NBHW], 2019a), similar guidelines in Norway and Finland state that the ASI *may* be used by professionals – a significantly weaker recommendation (Finnish Medical Society, 2026; Norwegian Directorate of Health, 2026). In Norway, the ASI is recommended regarding substance use problems in general but in Finland only regarding problems with illicit drugs. Further, the Danish health authority does not recommend ASI at all in its practice guidelines (Danish Health Authority, 2018; 2019)^1^.

Why, then, do Swedish health authorities reach such a dissimilar conclusion than authorities in other Nordic countries? And where is the evidence that supports the strong recommendation of the ASI? In this article, I argue that there is a lack of clear evidence that motivates the strong recommendation of the ASI in Swedish guidelines. My argument centers on the municipal social services sector where the ASI has gained most traction. I review the assessments underpinning the National Board of Health and Welfare's (NBHW) recommendation of the ASI, point out unclear links between presented evidence and recommendations, and call attention to research that is omitted from the guidelines. Finally, I discuss implications and possible ways forward regarding the social services’ assessment of clients with substance use problems.

## The ASI in Swedish National Practice Guidelines

The goal of practice guidelines is to produce systematic and transparent recommendations for practice that are based on the best available evidence (Guyatt et al., 2008). Basically, it is about weighing the pros and cons of alternative strategies and making the best recommendation. There are different ways to develop guidelines and one of the most common is Grading of Recommendations, Assessment, Development and Evaluation (GRADE), which is cited in the Swedish guidelines (NBHW, 2019a). In the GRADE system, the assessment is divided into two main components: “quality of evidence” and “strength of recommendations.” Although high quality evidence does not necessarily imply strong recommendations, and strong recommendations can be based on low quality evidence, guideline panels are urged to be transparent regarding how recommendations are reached and how other factors than evidence, such as expert opinion, values, and preferences, are weighed in (Guyatt et al., 2008, p. 925; GRADE, 2026).

In the Swedish guidelines, the ASI recommendation reads like this:

Healthcare and social services should:

Use the ASI to assess the support needs of people with alcohol or drug problems (priority 1). […] A key factor in the recommendation is that the instrument works well for assessing the need for support among people with alcohol and drug problems. (NBHW, 2019a, p. 27)

In the NBHW's definition, priority 1–3 means that the method/intervention “should be used,” priority 4–7 that it “may be used,” and priority 8–10 that it “may be used in exceptional cases” (NBHW, 2019a, p. 23).

The evidence and deliberations that underpin these recommendations is presented in a separate working document (NBHW, 2019b). In this document, the ASI is assessed in relation to two criteria: “technical quality” and “usability” (NBHW, 2019b).

### Technical Quality

Technical quality is divided into “reliability,” “validity,” and “relevant Swedish norms.” Reliability and validity concern measurement quality, which in the case of the ASI refers to how well the composite scores and interviewer severity ratings in each functional domain measure what they set out to measure. Reliability refers to the agreement of measurement between different professionals and between measurements over time regarding the same client. Validity refers to whether the ASI actually measures what it is supposed to measure, which can be studied by comparing it to external criteria, whether different items relating to a certain domain actually capture the same phenomenon, and whether measures can predict future events (e.g., if alcohol problem severity predicts future alcohol problems). The criterion “relevant Swedish norms” concerns whether there are available ASI data for a normal population, which can provide a baseline for evaluating and interpreting ASI scores in professional practice.

The panel responsible for outlining these guidelines bases its assessment on 13 studies that examine the validity and reliability of the ASI, and one study that covers “relevant Swedish norms.” On a four-point scale, the panel awards the ASI the second-highest grade, which in their own terms warrants the conclusion that “support for technical quality should be supplemented” (NBHW, 2019b, p. 209; 225). This means that the ASI does not live up to the standards of highest grade (i.e., “technical quality is good”). According to the panel, this is due to inconsistent results and lack of data on validity in the alcohol domain, and lack of data on both validity and reliability in the illicit drug domain.

### Usability

In the previously mentioned working document, usability concerns whether there is an available Swedish manual and the quality of this manual. For this criterion, no empirical studies are reviewed. It is simply stated that a Swedish manual exists and that it is of sufficient quality (NBHW, 2019b, p. 209; 225).

## Unclear Link Between Evidence and Recommendations

In this section, I critically review the assessments underpinning the guideline panel's recommendations. As in the previous section, my review focuses on two main criteria: technical quality and usability.

### Technical Quality

As regards technical quality, there are no obvious shortcomings concerning the studies that are included as a basis for recommendation. My main critical remarks concern the low-quality evidence of included studies and flaws in the available normative clinical data.

The first remark is that the guideline panel fails to address that the quality of evidence is low because of weak study designs. According to the GRADE handbook, which the NBHW purportedly follows, so called diagnostic intervention studies are considered to provide the best evidence when it comes to assessment instruments (GRADE, 2026, chapter 7)^2^. In this design, alternative assessment strategies are compared and evaluated examining outcomes that are directly important to clients (e.g., substance use and related problems). In this way, it is possible to determine whether one assessment strategy leads to better client-important outcomes than another. In the case of the ASI, no such studies are available (Becker et al., 2023, p. 53) and the guideline panel therefore bases its recommendation on other kinds of studies, predominantly studies on validity and reliability. According to the GRADE handbook, this reflects rather weak study designs as basis for practice recommendations. While the validity and reliability of measures are important aspects regarding the production quantitative data, they are not necessarily so for practice. Of greater relevance for practice is whether a particular assessment method leads to better assessments of suitable interventions for clients, which, in turn, should lead to better client outcomes. This is best studied using a diagnostic intervention study design.

The absence of such studies in the case of the ASI means that we cannot be certain that the ASI is better for clients than other assessment strategies. From the available evidence, we only know that the ASI is fairly good at measuring what it sets out to measure. In the absence of available diagnostic intervention studies, GRADE advises guideline panels to make inferences about the likely impact of assessment strategies on patient-important outcomes (GRADE, 2026). However, such inferences are neither presented in the NBHW's guidelines nor in their working document.

My second remark is related to flaws in the available normative clinical data. As the guideline panel mentions, there are no available ASI data regarding a representative Swedish normal population. In the absence of such data, the panel refers to a report which aims to establish clinical norm data based on a national database of ASI interviews conducted in routine social services and healthcare (Armelius et al., 2009). Based on this report, the guideline panel concludes that “The information on norm data is of a sufficiently high standard” (NBHW, 2019b, p. 209; 225). This conclusion is reached despite the fact that the authors of the report themselves point to serious issues concerning the representativity of the data. These clinical norm data are not based on a systematic sampling strategy but consist of available data from a small share (50 of 290) municipalities and one out of 20 regions that contributed to the database. Moreover, on a local clinical level, the ASI is by no means used with every client or patient, which raises serious questions about the representativeness of this clinical norm data (Armelius et al., 2009). Given that the main function of normative data is to provide a representative baseline for evaluating and interpreting ASI scores in professional practice, this material is not sufficient.

In sum, then, the available evidence regarding technical quality and the empirical basis for the panel's deliberations fail to provide strong arguments for recommending the ASI at the highest priority.

### Usability

When it comes to usability, the main problem is that the guideline panel does not take into account all relevant evidence. Their assessment is based purely on the ASI manual itself, not on how it is used in practice. A manual can be of very high quality, giving clear instructions and so forth, but still be of limited use in practice. Usability, or feasibility as it is often termed in the international literature, is to a large extent an empirical matter that can be studied using different research designs (Bowen et al., 2009).

As the ASI is recommended for use in the Swedish social services, it has also been the subject of empirical research. This research provides evidence on the usability of the ASI, which indicates that it is not used as intended. An evaluation of guideline compliance shows that the ASI is used in “assessment of a majority of clients” in 67% of the Swedish municipal social services (88 of the 131 municipalities that were studied; NBHW, 2024). Based on a review of social service cases in Stockholm city, preliminary findings show that the ASI was used in only 12% of the cases during the past year (Ekendahl, 2026). These low shares point towards limited usability of the ASI in Swedish social services.

Among studies that more directly examine the usability of the ASI in Swedish social services, only one presents findings that clearly support its usability (Engström, 2005). The study compared two groups of clients who had participated in an assessment session with or without ASI and found no differences regarding therapeutic alliance or negative experiences. However, the clients’ feeling of own competence was rated lower in the ASI group.

Other studies that examine the use of the ASI identify problems and concerns in three areas: comprehensiveness and time demands, lack of suitability for common client groups, and inflexibility. Regarding the first area, social workers find it cumbersome to use the ASI in practice (Alexanderson, 2006; Björk, 2013, 2017, 2026; Wikström & Lindberg, 2006), primarily due to its comprehensiveness and time demands. As noted above, intake and follow-up ASI interviews comprise 180 and 140 questions respectively, which take time and can be exhausting for clients as well as practitioners. These interviews also require additional administrative tasks, such as registering answers in a specific IT system. Regarding lack of suitability, clients, especially those with co-occurring mental health problems, find it difficult to respond to a multitude of thorough questions, and to recall details about what happened during the previous 6 months or earlier in life (Abrahamson & Tryggvesson, 2009; Björk, 2013; Engström & Armelius, 2005; Wikström & Lindberg, 2006). The inflexibility of the ASI refers to its standardized format, with predefined questions and response alternatives that do not fully capture clients’ own descriptions of problems and needs (Björk, 2013, 2017, 2026). Overall, social workers rarely find that the ASI provides meaningful information that they can use in their direct client work (Björk, 2013, 2017, 2026).

Although this research may be graded as fairly low quality, especially because most studies were not designed specifically to study usability or feasibility, I consider that it can and should inform the NBHW's assessment of ASI usability. This research is predominantly qualitative and the original GRADE system does not provide guidance concerning how to assess such studies. However, it is well known that qualitative studies are particularly suited for research questions that address aspects of usability (Petticrew & Roberts, 2003) and there is a separate GRADE system that focuses on qualitative evidence, the GRADE-CERQual (Confidence in the Evidence from Reviews of Qualitative Research; Lewin et al., 2018). Given that the research on the usability of the ASI in the Swedish social services is scarce but consistent when it comes to demonstrating a lack of usability, I draw a completely different conclusion than that of the guideline panel, namely that the usability of the ASI is far from established.

## Discussion

In this article, I question whether the evidence available for ASI's technical quality and usability really speaks in favor of strongly recommending its use in the Swedish social services. Given that national practice guidelines should provide systematic and transparent recommendations, this gap between evidence and recommendation is remarkable. According to the GRADE handbook, low quality evidence can result in strong recommendations, but, in such cases, guideline panels should clearly present what arguments are factored into assessments and deliberations (GRADE, 2026; Guyatt et al., 2008). At present, however, no such arguments are put forward.

I can only speculate on what factors, other than available evidence, that may have influenced the guideline panel's recommendation. Of note is that the guidelines are commissioned by the same agency that also owns the ASI, namely the NBHW. In a way, the NBHW is assessing its own product which should indicate a conflict of interest. Although the guideline panel is supposed to be independent from the NBHW, there might be a built-in bias towards producing favorable recommendations. Another potential factor concerns what might be called path dependence. A great deal of work and resources have been invested in the ASI: development of manuals and forms, ASI training programs, and a nationwide IT system supporting the administration, storage, analysis, and use of ASI data. Thus, there might be social pressure on the guideline panel to issue a positive recommendation that legitimizes the well-trodden path. A final potential factor is the lack of readymade alternatives to the ASI. Indeed, the ASI is the most empirically tested multi-dimensional assessment method within the field, and, compared to other methods, it stands out as having the strongest evidence-base. But this does not necessarily motivate a strong recommendation. Indeed, the lack of diagnostic intervention studies means that we cannot know for sure whether the ASI is better for clients than other assessment methods.

The guideline recommendation has resulted in a strong pressure on Swedish social services to use the ASI in practice. As an example, I have in my own research noticed that social workers’ use of the ASI is a factor in their individual salary setting. Coupled with a perceived lack of usability, this puts social workers in a difficult position. However, due to the ASI's air of “evidence-basedness,” many have not dared to voice their concerns and discontent. In light of the rather weak evidence for the ASI that I point to in this article, there might good reasons to ease the demands on social workers to use the ASI.

In addition to easing the demands to use the ASI, there is a need to develop new and more feasible assessment methods for the social services. Against this background, I am currently involved in a research program seeking to advance a new assessment and follow-up method for the social services. The starting point was to create a less extensive and more flexible assessment and follow-up method (compared to the ASI) that could be tailored to the complexities of various client groups that contact the social services. To ensure good feasibility, the method is developed in collaboration with experienced statutory social workers, social service managers, and quality developers at different levels. During the writing of this article, the method is being tested in social service practice. It will eventually be evaluated in terms of feasibility (clients’ and social workers’ experiences) and in terms of client outcomes.

It remains to be seen whether this method is a suitable replacement for the ASI. In the meantime, there are, in my view, strong arguments to downplay requirements of using ASI in Swedish social services.

## Notes

1. At present, no national practice guidelines specifically for the treatment of substance use problems exist in Iceland (Ministry of Health of Iceland, 2024).

2. GRADE focuses on diagnostic tests. While the ASI is not a diagnostic test, it is intended to serve a similar purpose, which is to support professionals to make the best decisions about interventions for their clients.
